# Delayed Post-hypoxic Leukoencephalopathy (DPHL)—An Uncommon Variant of Hypoxic Brain Damage in Adults

**DOI:** 10.3389/fneur.2018.00708

**Published:** 2018-08-27

**Authors:** Anne B. Beeskow, Moritz Oberstadt, Dorothee Saur, Karl-Titus Hoffmann, Donald Lobsien

**Affiliations:** ^1^Department of Pediatric Radiology, University Hospital Leipzig, Leipzig, Germany; ^2^Department of Neurology, University Hospital Leipzig, Leipzig, Germany; ^3^Department of Neuroradiology, University Hospital Leipzig, Leipzig, Germany

**Keywords:** delayed post-hypoxic leukoencephalopathy, DPHL, hypoxic brain damage in adults, white matter, ischemia, neuroradiology, CT, MRI

## Abstract

Delayed post-hypoxic leukoencephalopathy (DPHL) is an uncommon, potentially under-recognized, cause of hypoxia induced white matter injury. It characteristically follows a biphasic course: After an initial phase of altered neurologic status a recovery occurs which is then followed by a recurring phase of neurologic deterioration, typically 2–4 weeks after the initial event. At this time white matter changes can be identified on MRI, which are the hallmark of DPHL. The characteristics and the typical MR-imaging signs of DPHL are discussed in this case report.

## Introduction

Delayed post-hypoxic leukoencephalopathy (DPHL) is an uncommon, potentially under-recognized cause of hypoxia-induced white matter injury. DPHL typically occurs following a period of prolonged cerebral hypo-oxygenation, and follows a biphasic course. An initial period of altered mental status is followed by a dramatic recovery, often to initial baseline. Then, typically 2–4 weeks later, neurocognitive deterioration recurs (1). At this point in time, a characteristic leukoencephalopathy can be detected on MRI, which represents the diagnostic hallmark of DPHL.

## Case report

A 51-year-old woman was found comatose and hypotonic in her home. The patient was resuscitated and intubated on site and admitted to an external hospital. An opiate antagonization, because of suspected opiate intoxication (she was on a treatment of for chronic pain syndrome with fentanyl patches), did not show any effect on the patient's consciousness. She had a past medical history of arterial hypertension, obesity, sleep apnea syndrome, and depression.

A blood sample-laboratory analysis revealed rhabdomyolysis. Subsequently, the patient developed acute kidney- and liver failure, which led to immediate transfer to the intensive care unit of our hospital.

**Figure 1:**
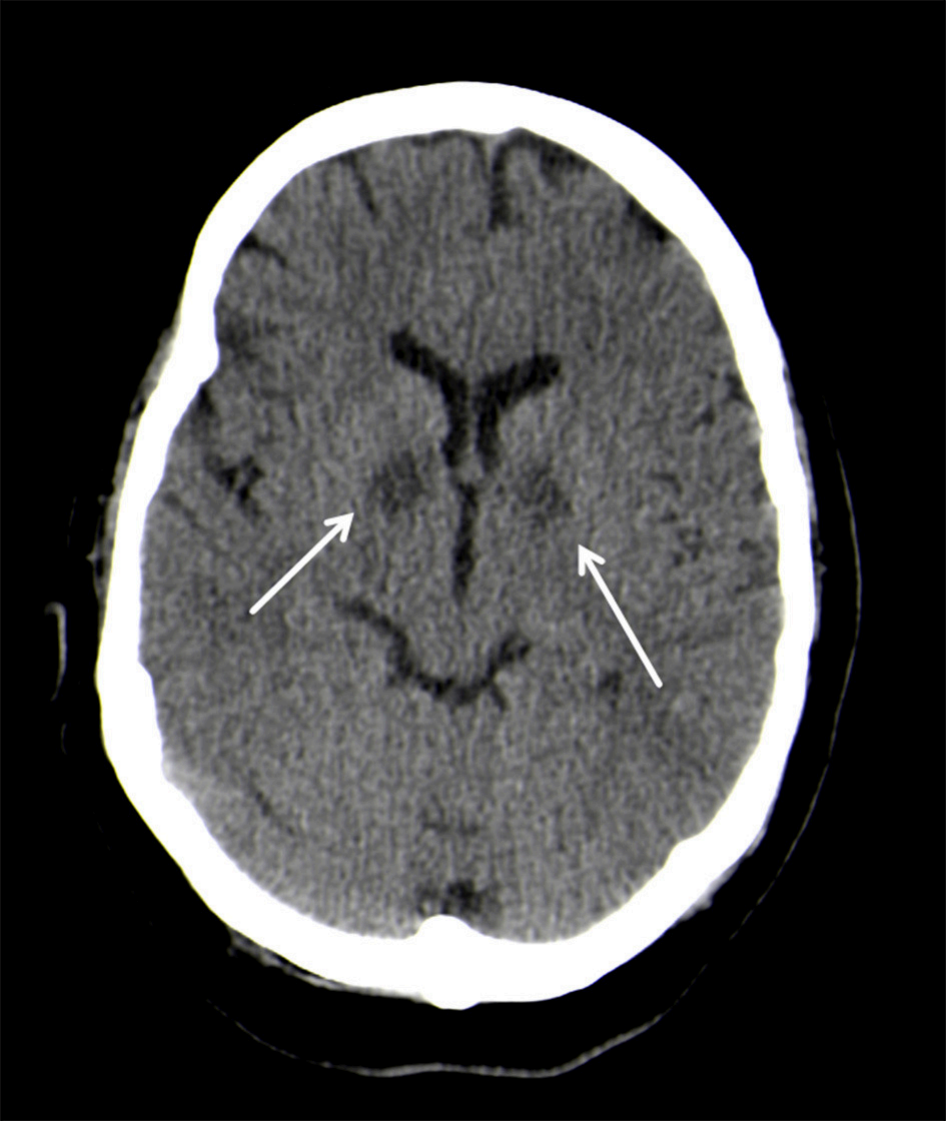
Bilateral, hypodense basal ganglia necrosis in unenhanced CT (arrows); Philips Ingenuity 5 mm.

On neurological examination, the patient presented with coma, but did not show any focal neurologic impairment. An unenhanced computed tomography (CT) of the head showed almost symmetrical bilateral hypointensities of the globus pallidus (Figure 1). These changes were interpreted as of primarily hypoxic origin, possibly caused by carbon monoxide (CO) poisoning, although there were no anamnestic indications supporting this assumption. A spinal tap showed no pathological findings of the cerebrospinal fluid (CFS). Four days after the initial event, the patient clinically improved and was cleared for extubation. The neurological examination thereafter was discreet with no focal neurological deficits and her mental status returned to normal.

**Figure 2:**
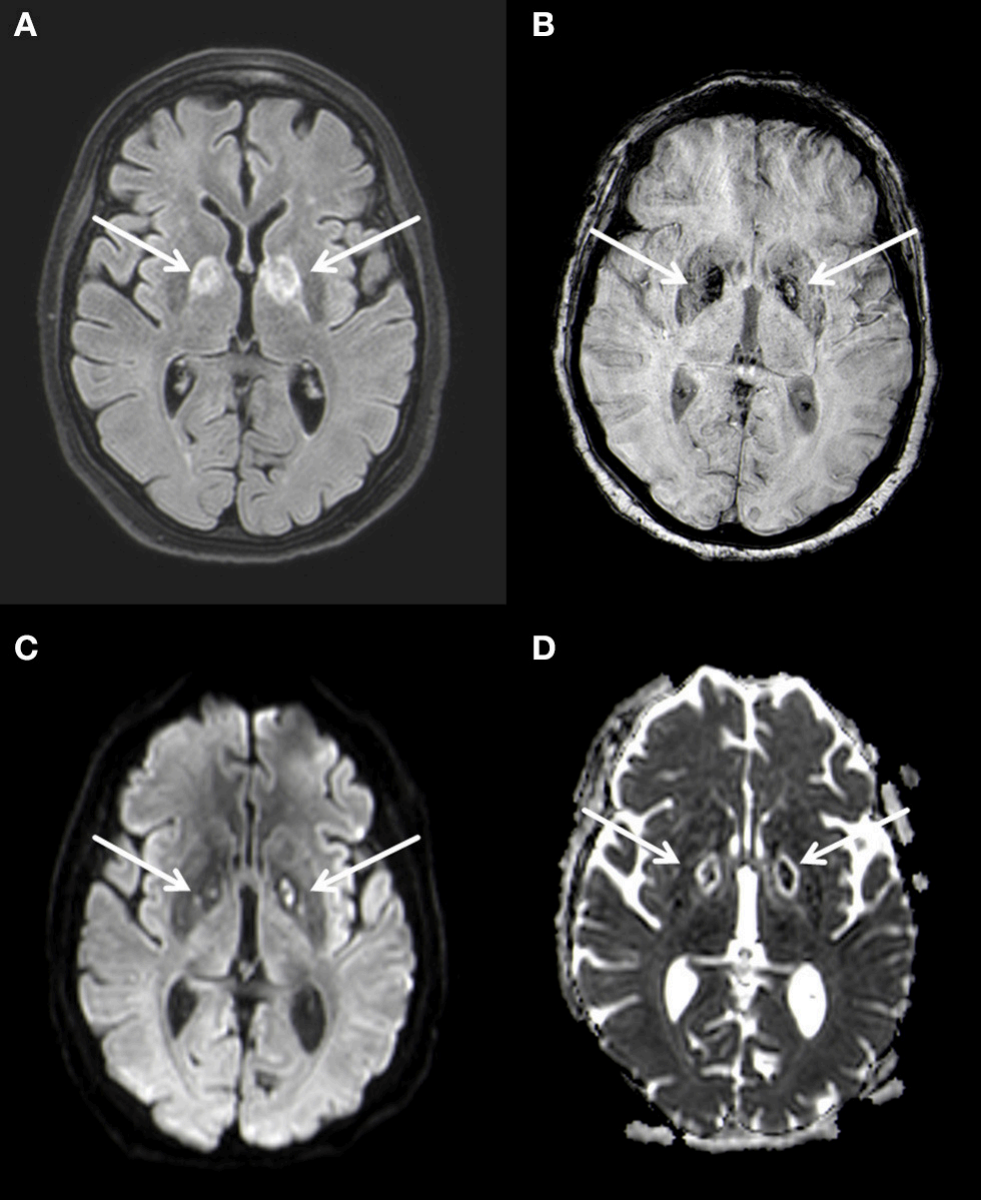
Bilateral basal ganglia necrosis with T2w hyperintense alterations **(A)** and hemoside deposits **(B)**. These changes are diffusion-disturbed **(C,D)**. 3 T Philips Ingenia, **(A)** FLAIR, **(B)** SWI, **(C)** b1000 image, **(D)** ADC map.

MRI of the brain 3 weeks after hospitalization confirmed the bilateral lesions of the globus pallidus seen on CT, characterized by restricted diffusion and FLAIR-hyperintense signal changes (Figure 2). At this time no leukoencephalopathy could be detected. These findings were again interpreted as of post hypoxic origin.

Approximately 3 weeks after the initial event, the patient developed progressive neuropsychiatric symptoms. First, she attracted attention with odd behavior (e.g., urinating into the rubbish bin or other patients' beds) and phases of agitation. Within a few days, the disturbance in behavior turned into a clinical picture dominated by reduced psychomotor activity and apathy, finally progressing into mutism. In addition, a novel increase in muscle tone with generalized rigidity and spontaneous myoclonus was observed.

**Figure 3:**
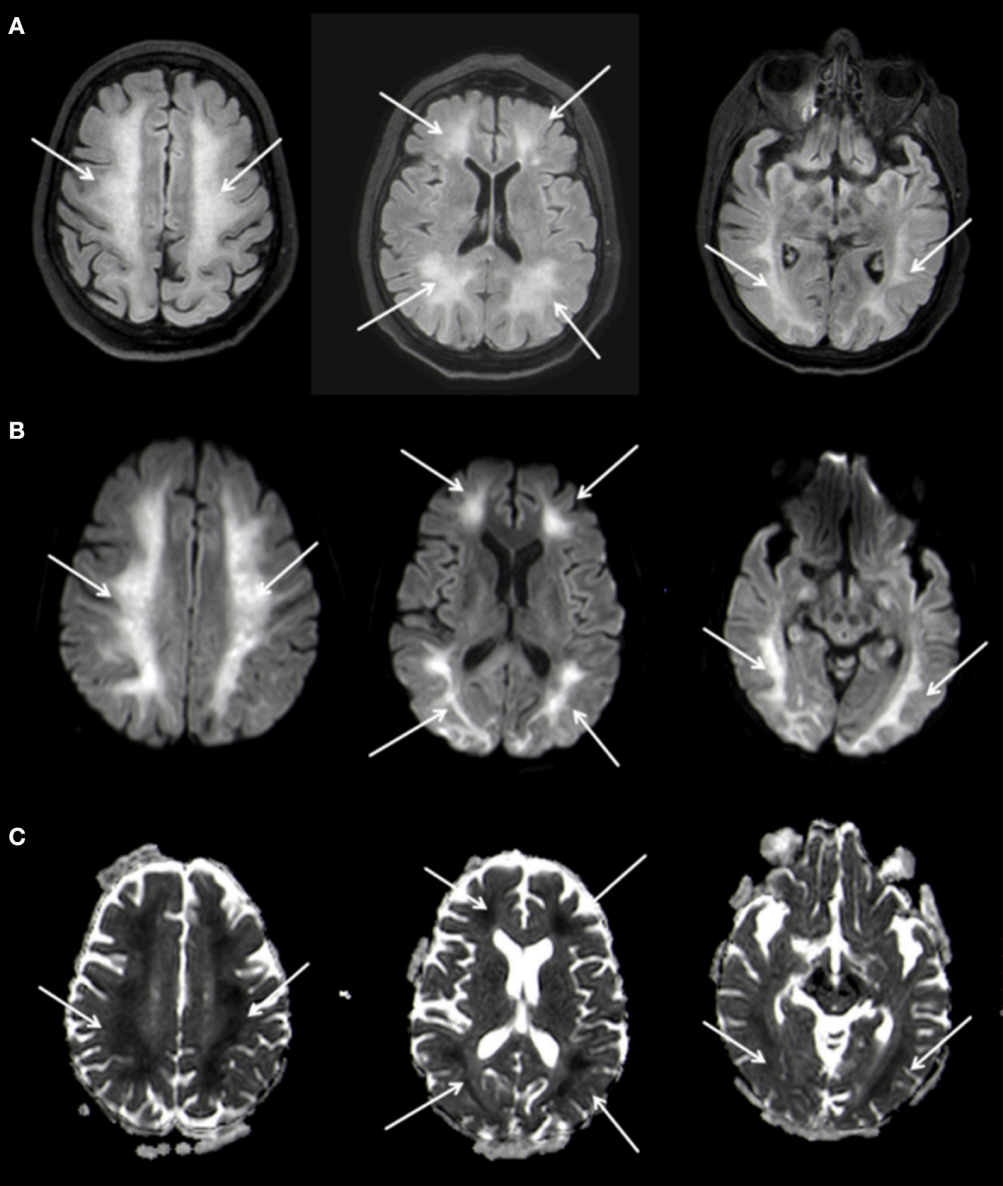
Planar T2w hyperintense signal changes of the entire white matter **(A)** with diffusion restriction **(B,C)** 3 T Philips Ingenia, **(A)** FLAIR, **(B)** b1000 Figure, **(C)** ADC Map.

A follow-up MRI-scan of the brain 5 weeks after the initial event showed, in addition to the known changes of the basal ganglia, a symmetrical, extensive increased FLAIR-signal with correlating marked diffusion restrictions of the white matter, primarily in the fronto-parietal regions of both hemispheres (Figure 3). The cortex remained spared from these changes. Neither the brain stem nor the cerebellum showed any pathological changes on MRI. In conjunction with the clinical course the changes were diagnosed as DPHL.

**Figure 4:**
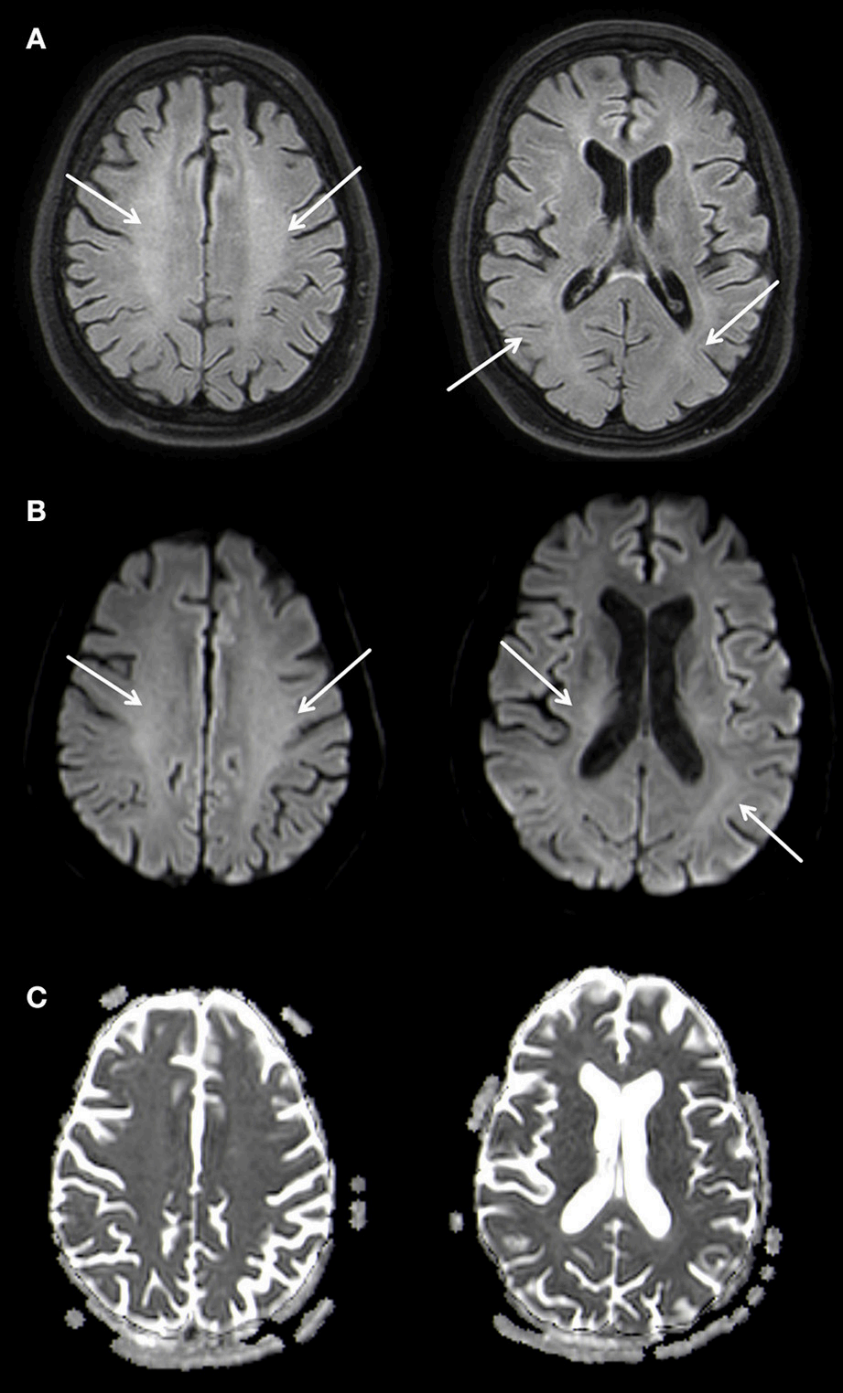
Slightly T2w hyperintense signal changes of the white matter **(A)** without diffusion restriction **(B,C)**, Atophy of the parenchyma with slightly wider inner CSF. 3 T Philips Ingenia, **(A)** FLAIR, **(B)** b1000 Figure, **(C)** ADC Map. Overall there is a marked regression of the imaging findings.

A supportive therapy followed. During the course of the inpatient stay, the patient's impairment remained unchanged and she was released into early neurological rehabilitation about 6 weeks after the initial event. A follow up MRI nine months later showed a marked regression of the leukoencephalopathy with a remaining faint hyperintensity predominantly in the parietal white matter of both hemispheres and a complete regression of the diffusion restriction. A slight symmetrical enlargement of the ventricles could be seen indicating a mild atrophy (Figure 4).

## Discussion

DPHL is a rare and probably underrecognized form of hypoxic brain damage (1). The combination of the characteristic clinical symptoms and development and the imaging findings on cranial-MRI are key to establishing the diagnosis (1). After an initial hypoxic event with neurologic deterioration the patient typically improves until neurological deterioration reoccurs, about 3 weeks (2–40 days) after the initial event. Two forms of clinical manifestation, based on the leading symptoms during the second phase, are described (2, 3): An akinetic-mute form, as seen in our case, is clinically represented by bizarre behavior, faulty actions and psychomotor retardation progressing into mutism. The second form resembles symptoms of parkinsonism.

The typical imaging pattern of DPHL is best detected on MRI. After a latency of about 3 weeks new T2-hyperintense changes of the white matter can be seen, which are typically homogeneously configured and affect large areas of white matter of both hemispheres in a symmetric distribution. These areas typically show a marked diffusion restriction, often to the same extends as the T2-hyperintense lesions. Characteristically, the cortex and U-fibers, cerebellum and the brain stem are spared. Also, in most case series the diffusion restriction seen in DPHL seems to last substantially longer in comparison to the diffusion restriction seen in ischemic stroke (4). This pattern makes it possible to differentiate DPHL from competing diagnoses others than hypoxic brain damage, e.g., generalized brain edema after global hypoxia, toxic brain damage, as seen in heroin inhalation syndrome, metronidazole, or methotrexate induced leukoencephalopathy, as well as posterior reversible encephalopathy syndrome (PRES) (2). These diagnoses have in common that they typically show diffusion restrictions which extend beyond the white matter into the cortical rim.

There is no documentation of a contrast enhancement in cases of DPHL in the literature and this was also not seen in our case. In cases where MR-spectroscopy was performed, investigated areas showed a low N-acetylaspartate peak, indicating neurons loss, an increased choline peak as a consequence of the demyelination process, and an increased lactate peak was also described in DPHL patients, suggesting a shift from aerobic to anaerobic metabolism (5). However, the imaging findings most important for the diagnosis DPHL are the changes on T2 weighted and diffusion-weighted MR-images.

The pathomechanism of DPHL has not yet been fully understood. Myelin-sheath damage is suspected, which might be caused by a prolongation of moderate hypooxygenation, due to a dysfunction of the ATP-dependent enzyme that causes myelin secretion. Myelin secretion occurs every 19 to 22 days, which correlates with the delayed occurrence of symptoms of 2–40 days (on average 23 days) (1). Another explanation might be the delayed apoptosis of oligodendrocytes (6). DPHL is described mainly in the context of carbon monoxide intoxication (in up to 3% of the cases), however various other causes of hypoxia, such as drug overuse, cardiac arrest, strangling or seizures are reported to provoke DPHL as well (1, 4, 7).

The therapy is mainly supportive. The benefit of hyperbaric oxygen therapy in case of a DPHL with CO-intoxication is controversial (6). The prognosis of DPHL varies but is generally considered to be good, with a majority of patients who survived the second phase of deterioration showing significant recovery (2, 5). A complete neurological recovery was described in individual cases (8), however, lasting frontal-executive deficits in neuropsychological testing are common (5).

In conclusion, DPHL is a rare and probably underrecognized form of prolonged hypoxia progression and combination of the characteristic biphasic clinical course with bilateral MRI signal changes limited to the white matter are considered pathognomonic. However, due to the relatively late manifestation, the typical symptoms and imaging signs, it might be overlooked by the unaware clinician. To evaluate the course and impact of DPHL further, studies following patient's clinical development and imaging features after hypoxic events might be warranted.

## Ethics statement

The written informed consent of the Patient is available.

## Author contributions

All authors listed have made a substantial, direct and intellectual contribution to the work, and approved it for publication.

### Conflict of interest statement

The authors declare that the research was conducted in the absence of any commercial or financial relationships that could be construed as a potential conflict of interest.
